# Mediation Analysis Demonstrates That *Trans*-eQTLs Are Often Explained by *Cis*-Mediation: A Genome-Wide Analysis among 1,800 South Asians

**DOI:** 10.1371/journal.pgen.1004818

**Published:** 2014-12-04

**Authors:** Brandon L. Pierce, Lin Tong, Lin S. Chen, Ronald Rahaman, Maria Argos, Farzana Jasmine, Shantanu Roy, Rachelle Paul-Brutus, Harm-Jan Westra, Lude Franke, Tonu Esko, Rakibuz Zaman, Tariqul Islam, Mahfuzar Rahman, John A. Baron, Muhammad G. Kibriya, Habibul Ahsan

**Affiliations:** 1 Department of Public Health Sciences, The University of Chicago, Chicago, Illinois, United States of America; 2 Comprehensive Cancer Center, The University of Chicago, Chicago, Illinois, United States of America; 3 University of Groningen, University Medical Center Groningen, Groningen, The Netherlands; 4 Estonian Genome Center, University of Tartu, Tartu, Estonia; 5 UChicago Research Bangladesh, Mohakhali, Dhaka, Bangladesh; 6 Research and Evaluation Division, BRAC, Dhaka, Bangladhesh; 7 University of North Carolina, Lineberger Comprehensive Cancer Center, Chapel Hill, North Carolina, United States of America; 8 Department of Medicine The University of Chicago, Chicago, Illinois, United States of America; 9 Department of Human Genetics, The University of Chicago, Chicago, Illinois, United States of America; Stanford University, United States of America

## Abstract

A large fraction of human genes are regulated by genetic variation near the transcribed sequence (*cis*-eQTL, expression quantitative trait locus), and many *cis*-eQTLs have implications for human disease. Less is known regarding the effects of genetic variation on expression of distant genes (*trans*-eQTLs) and their biological mechanisms. In this work, we use genome-wide data on SNPs and array-based expression measures from mononuclear cells obtained from a population-based cohort of 1,799 Bangladeshi individuals to characterize *cis*- and *trans*-eQTLs and determine if observed *trans*-eQTL associations are mediated by expression of transcripts in *cis* with the SNPs showing *trans*-association, using Sobel tests of mediation. We observed 434 independent *trans*-eQTL associations at a false-discovery rate of 0.05, and 189 of these *trans*-eQTLs were also *cis*-eQTLs (enrichment P<0.0001). Among these 189 *trans*-eQTL associations, 39 were significantly attenuated after adjusting for a *cis*-mediator based on Sobel P<10^-5^. We attempted to replicate 21 of these mediation signals in two European cohorts, and while only 7 *trans*-eQTL associations were present in one or both cohorts, 6 showed evidence of cis-mediation. Analyses of simulated data show that complete mediation will be observed as partial mediation in the presence of mediator measurement error or imperfect LD between measured and causal variants. Our data demonstrates that *trans*-associations can become significantly stronger or switch directions after adjusting for a potential mediator. Using simulated data, we demonstrate that this phenomenon is expected in the presence of strong *cis*-*trans* confounding and when the measured *cis*-transcript is correlated with the true (unmeasured) mediator. In conclusion, by applying mediation analysis to eQTL data, we show that a substantial fraction of observed *trans*-eQTL associations can be explained by *cis*-mediation. Future studies should focus on understanding the mechanisms underlying widespread *cis*-mediation and their relevance to disease biology, as well as using mediation analysis to improve eQTL discovery.

## Author Summary

Expression quantitative trait locus (eQTL) studies have demonstrated that human genes can be regulated by genetic variation residing close to the gene (*cis-*eQTLs) or in a distant region or on a different chromosome (*trans*-eQTLs). While *cis*-eQTL variants are likely to affect transcription factor binding or chromatin structure, our understanding of the mechanisms underlying *trans*-eQTLs is incomplete. We hypothesize that a substantial fraction of *trans*-eQTLs influence expression of distant genes through mediation by expression levels of a *cis*-transcript. In this paper, we use genome-wide SNPs and expression data for 1,799 South Asians to identify cis- and trans-eQTLs and to test our hypothesis using Sobel tests of mediation. Among 189 observed trans-eQTL associations, we provide evidence of *cis*-mediation for 39, 6 of which show mediation in an independent European cohort. We used simulated data to demonstrate that complete mediation will be observed as partial mediation in the presence of mediator measurement error or imperfect LD between measured and causal variants. We also demonstrate how unobserved confounding variables and incorrect mediator selection can bias mediation estimates. In conclusion, we have identified *cis*-mediators for many *trans*-eQTLs and described a mediation analysis approach that can be used to validate, characterize, and enhance discovery of *trans*-eQTLs.

## Introduction

The development of technologies that enable high-throughput, genome-wide measurement of single nucleotide polymorphisms (SNPs) and mRNA transcripts have enabled researchers to comprehensively examine the effects of human genetic variation on gene expression. Genome-wide studies of expression quantitative trait loci (eQTLs) have been conducted using a wide-array of RNA sources, including lymphoblastoid cells lines [1]–[4], whole blood [5], monocytes [6], [7], B-cells [7], liver cells [8], [9], and breast cancer tumor cells [10]. These studies consistently demonstrate that a large fraction of human genes (perhaps all genes) are regulated by variants near the transcribed sequence, typically referred to as *cis*-eQTLs (or *cis*-eSNPs).

Less is known regarding the effects of genetic variation on expression of distant genes and genes residing on other chromosomes (i.e., *trans*-eQTLs). Identifying *trans*-eQTLs should provide insight into the mechanisms of gene regulation, including mechanisms relevant to disease-associated variants and human disease biology. *Trans*-eQTLs are more difficult to identify than *cis*-eQTLs because *trans* effects are generally weaker than *cis* effects and because a huge number of tests must be conducted to comprehensively search the genome for *trans*-eQTLs, resulting in the use of stringent significance thresholds. Thus, large studies are needed for *trans*-eQTL identification. Several such studies (>1,000 participants) have focused on identifying *trans*- eQTLs, and these have typically used white blood cells as an RNA source [5], [6], [11]. In early *trans-*eQTL studies, the proportion of *trans*-eQTLs replicated across studies was quite low, much lower than *cis*-eQTLs [9], but a recent study demonstrates that most *trans*-eQTLs replicate when very large sample sizes are used. [11]

While the biological mechanisms underlying *trans*-eQTLs are largely unknown, it is likely that many *trans*-eQTLs are also *cis*-eQTLs, and it is the *cis*-transcript that affects the expression of a *trans*-gene; however, no prior *trans*-eQTL studies have systematically assessed evidence for *cis*-mediation among identified *trans*-eQTLs. While replication in independent samples is the gold standard method for validating *trans*-eQTLs, we propose that documenting *cis*-mediation can provide additional evidence that an observed *trans*-association is a true *trans*-eQTL and a potential biological explanation/mechanism for the observed *trans*-eQTL.

In this work, we describe *cis*- and *trans*-eQTL associations using data on genome-wide SNPs and genome-wide RNA transcripts (extracted from mononuclear cells) for 1,799 Bangladeshi adults. For SNPs observed to be *trans*-eQTLs, we use a mediation analysis approach to assess evidence that the observed *trans*-eQTL associations are mediated by measured transcripts that are in *cis* with the SNP showing a *trans*-association. We observe evidence of mediation for a substantial fraction of the *trans*-eQTLs observed in our data and replicate several of our mediation signals in an independent sample, suggesting that many observed *trans*-eQTLs are due to mediation by expression levels of *cis*-transcripts in the vicinity of the *trans*-eQTL. These observations can be used to enhance our understanding of regulatory mechanisms and our ability to identify *trans*-eQTLs.

## Results

### Genome-wide eQTL analysis

We conducted genome-wide *cis*-eQTL analysis using data on 1,016,489 genotyped and imputed SNPs and 22,973 expression probes (corresponding to 16,006 genes) measured for 1,799 Bangladeshi individuals, using DNA extracted from whole blood and RNA extracted from peripheral blood mononuclear cells (PBMCs). For both SNP and probe data, stringent quality control (QC) measures were implemented to eliminate false positive associations (see methods). Results of genome-wide eQTL analyses are summarized in **Table 1**. At a genome-wide false-discovery rate (FDR) of 0.05 (P<2.2×10^−3^), we observed that 15,570 out of 22,973 expression probes (68%) and 11,827 out of 16,006 unique genes (74%) show evidence of a *cis*-eQTL in this population.

**Table 1:** Summary of *cis*- and *trans*-eQTL signals identified in genome-wide^1^ analyses using a false discovery rate (FDR^2^) of 0.05.

	*Cis*-eQTL analysis (SNP and probes <1 Mb apart)	*Trans*-eQTL analysis (SNPs and probes>10 Mb apart)
**Genome-wide SNPs (n = 1,016,489)**		
Tests conducted	16,189,390	32,168,439,253
Significant SNP-probe pairs^3^	628,442	4,234
Significant eQTL SNPs^3^	303,974	3,046
Significant eQTL probes	15,570	427
Significant eQTL genes	11,827	414
**Trait-associated SNPs (n = 1,930)**		
Tests conducted	45,414	60,465,173
Significant eQTL SNPs	950	67
% GWAS SNPs that are eQTL SNPs	49.2%	3.5%
Enrichment P^4^	2.8×10^−42^	1.6×10^−12^

1 Analyses were conducted using 31,853 expression probes for *trans* analysis, 22,973 probes for *cis* analysis and 1,016,489 genotyped and imputed SNPs.

2 A FDR of 0.05 corresponded to P-value thresholds of 2.2×10^−3^ for the *cis*-eQTL analysis and 8.4×10^−9^ for *trans*-eQTL analysis.

3 Counts include SNPs in LD.

4 Methods for calculation enrichment P-values are provided in the methods section.

In the genome-wide *trans*-eQTL analysis using an FDR of 0.05 (P<8.4×10^−9^), we observed 427 significant expression probes, corresponding to 414 unique genes (Table 1). Among these probes, there were 434 unique eQTL associations (i.e., unique probe and unique *trans*-eQTL region), corresponding to 419 unique *trans*-eQTL associations at the gene-level (Table S1 and Figure S1). There were 26 examples of a single variant (or variants in strong LD) showing association with multiple unique *trans* genes and 11 examples of a gene affect by multiple *trans*-eSNPs located in different regions of the genome. We observe many *trans*-eQTLs reported in prior studies, including the monocyte-specific master regulator at the LYZ locus on chromosome 12 identified by Fairfax (rs10784774) [7], the multiple *trans*-effects of variation type 1 diabetes region 12q13.2 described by Fehrmann and Fairfax [5], [7], and the lupus SNP rs7917014 (tagged by rs4917014 in our data) association with CLEC4C, CLEC10A, IFIT1, and other genes highlighted by Westra [11].

### Enrichment analysis for eQTLs

Consistent with findings from previous studies [5], [11], [12], SNPs known to be associated with human traits (1,930 unlinked SNPs with P<5×10^−8^ in the NHGRI GWAS catalog) were more likely to show association with local gene expression (P = 2.8×10^−42^) (Table 1 and Figure S2). Similarly, we observed that trait-associated SNPs are more likely to be *trans*-eQTLs (enrichment P = 1.6×10^−12^), with 67 trait-associated SNPs (∼3.5%) showing strong evidence of *trans*-association (Table 1 and Figure S3).

In addition, we show that *trans*-eSNPs are more likely to be *cis*-eSNPs than randomly-selected SNPs (enrichment P<0.001, consistent with Westra et al.[11], Figure S4), with ∼45% of lead *trans*-eSNPs identified as *cis*-eSNPs, suggesting that the effect of many *trans*-eQTL effects may be mediated by measured transcripts regulated in *cis* by the causal *trans*-eSNP. At *trans*-eQTL P-value thresholds of 10^−15^, 8.4×10^−9^, 10^−7^, the percentages of *trans*-eQTLs (lead SNPs) that were also associated with *cis*-transcripts were 62% (45 out of 73), 44% (189/434), and 31% (781/2,536), respectively (Table 2). Genes that lie <30kb from lead trans-eSNPs are more likely to be associated with SNPs in *cis* (76.2% of probes) as compared to all transcripts (67.8% of probes) (P = 1.6×10^−4^).

**Table 2:** Percent of *trans*-eQTL signals^1^ showing evidence of *cis*-mediation according to LD between the lead *trans*-eSNP and the lead *cis*-eSNP and the P-threshold for *trans*-eQTL analysis.

	*Trans*-eQTL associations with a potentially mediating *cis*-transcript			*Trans* e-QTL associations lacking an associated *cis*-transcript
P-threshold for *trans* analysis	r^2^between lead *trans*- and lead *cis*-eSNPs^2^	*Trans*-eQTL associations	% Mediated^4^	
<10^−15^	<0.5	11	9.1	
	0.5–0.9	8	50.0	
	≥0.9	26	53.9	
	**Total**	**45**	**42.2**	**28**
<8.4×10^−9,^ 3	<0.1	34	0	
	0.1–0.5	47	12.8	
	0.5–0.9	36	25.0	
	≥0.9	72	36.1	
	**Total**	**189**	**21.7**	**245**
<10^−7^	<0.1	275	1.8	
	0.1–0.3	131	6.1	
	0.3–0.5	72	5.6	
	0.5–0.7	69	8.7	
	0.7–0.9	78	11.5	
	≥0.9	156	21.8	
	**Total**	**781**	**8.5**	**1,755**

1 The *trans*-eQTL signals presented are probe-level signals.

2 r^2^ is a measure of LD between the lead SNP for the *trans*-eQTL signal and the lead *cis*-eSNP for the potentially mediating transcript (i.e., probe).

3 P-threshold of 8.4×10^−9^ corresponds to the significance threshold used in Table 1 (FDR of 0.05). At this threshold, the 189 signals with a *cis*-probe and the 245 signals without a *cis*-probe constitute the 434 eQTL signals (comprised of 414 unique genes reported in Table 1).

4 Mediated is defined as *trans*-eQTL signals with a “mediation proportion” estimate>0 and Sobel P<10^−5^.

### Evidence for *cis*-mediation among a subset of *trans*-eQTLs

**Figure 1:**
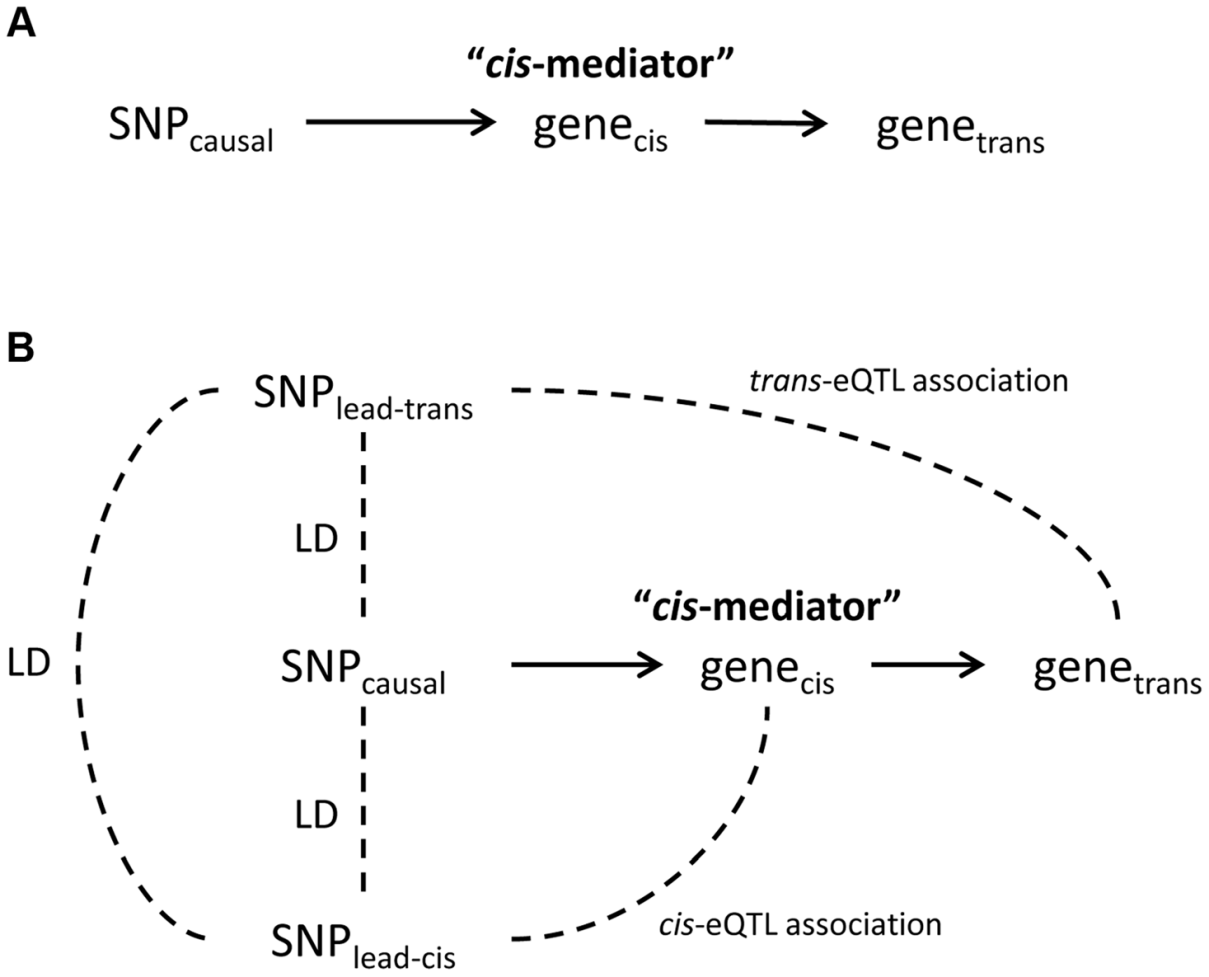
Theoretical framework for *cis*-mediation of *trans*-eQTLs. Panel (A) shows a causal diagram, in which a causal variant (SNP_causal_) affects expression of a *cis*-transcript (gene*_cis_*) which in turn affects expression of a distant gene (gene*_trans_*). When SNP_causal_ is measured and is the strongest associated SNP (i.e., “lead SNP”) for both the *trans*- and *cis*-eQTL association signals, no other SNPs are involved in mediation analysis. Panel (B) shows the causal diagram underlying an eQTL mediation analysis when the causal variant is unmeasured or is not the lead SNP for both the *trans*- and *cis*-eQTL association signals. Thus, the lead SNP for gene*_cis_* and gene*_trans_* may be different, and are noted here as SNP_lead-*cis*_ and SNP_lead-*trans*_, respectively. Solid lines represent causal effects, and dotted lines represent non-causal correlation, including linkage disequilibrium (LD).

**Figure 2:**
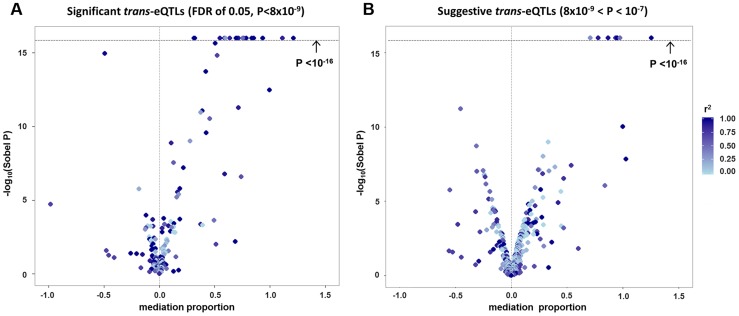
Strong evidence of *cis*-mediation is detected only when the lead *trans*-eSNP and the lead *cis*-eSNP for the mediating transcript are in strong LD. The proportion of a *trans*-eQTL mediated by a *cis*-transcript (i.e., the “mediation proportion) is plotted against the negative log_10_ of the Sobel P value for mediation for “FDR-significant” *trans*-eQTLs (panel A) and for “suggestive” *trans*-eQTLs (panel B). The plot is truncated at a Sobel P value of 10^−16^. Two outliers with a “mediation proportion”>2 have been excluded from panel A.

Among the 434 unique probe-level *trans*-eQTL associations, there were 189 for which the strongest associated SNP (i.e., “lead *trans*-eSNP”) was also associated with at least one local *cis*-transcript (based on genome-wide significance in the *cis*-eQTL analysis), representing a potential mediator of the *trans*-eQTL association (Fig. 1A). For these 189 *trans*-eQTL associations we performed mediation analysis (see methods) and obtained Sobel P-values for mediation as well as an estimate of the proportion of the *trans*-eQTL effect that is mediated by a *cis*-transcript (i.e., the proportion reduction in the magnitude of the beta coefficient after adjusting for the potential mediator: (β - β_adj_)/β). These are plotted in Fig. 2A, which shows an excess of “mediation proportion” estimates that are greater than zero. At a Sobel P threshold of 10^−5^ (based on visual inspection of Fig. 2A) 39 *trans*-eQTL associations (21%) were significantly reduced in magnitude after adjusting for a *cis*-transcript, suggesting that the measured transcript is the primary mediator of the *trans*-eQTL effect (Table S2). Extending our analysis to the 592 *trans*-eQTL associations that did not pass the FDR threshold, but had a P<10^−7^ and a potential *cis*-mediator, we observed evidence of mediation for seven additional *trans*-eQTLs with Sobel P <10^−16^ and one *trans*-eQTL that changed direction after adjustment for the *cis*-transcript (Fig. 2B and Table S2), suggesting that mediation analysis can be used to enhance *trans*-eQTL discovery.

Evidence for *cis*-mediation was stronger when LD between the lead *trans*-SNP and the lead *cis*-eSNP was high (Table 2 and Fig. 2), as expected under the mediation hypothesis, which implies the observed *cis*- and *trans*-eQTL associations are due to the same causal variant (Fig. 1B). Evidence for *cis*-mediation was also stronger for *trans*-eQTLs with smaller P-values (Table 2), suggesting that mediation is a characteristic of true positives and that power for detecting mediation is higher for stronger *trans*-eQTLs. Similarly, at less stringent thresholds, a smaller percentage of *trans*-eQTL variants show association with a *cis*-transcript (Table 2), suggesting that observed *trans*-eSNPs that are not associated with a nearby transcript are, on average, less likely to be true *trans*-eQTLs.

**Figure 3:**
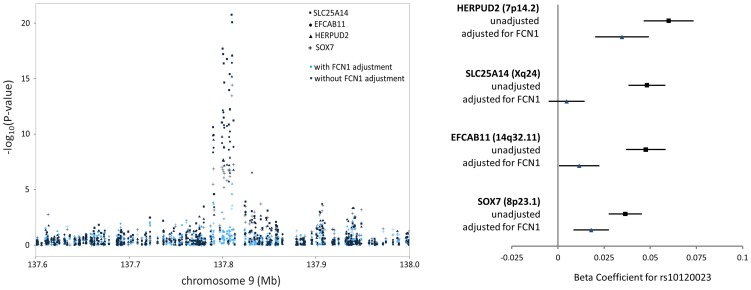
FCN1 expression is the primary mediator of the *trans*-eQTL at the 9q34.3 white blood cell subtype locus, affecting expression of four genes in *trans*. The P-values (left) and beta coefficients (right) for four *trans*-eQTL associations in the FCN1 region are reduced in significance after adjusting for FCN1 expression.

Among the 39 *trans*-eQTL associations showing strong evidence of mediation (**Table S2**), seven *trans*-eQTLs were present more than once, represented by *cis*-mediators AK125871, GNLY, GATA2, TREML1, FCN1, RPS26, and RBPMS2. The *trans*-eSNPs showing mediation by GATA2 (3q21.3), FCN1 (9q34.3), and RPS26 (12q13.2) are also associated with white blood cell subtypes [13], systemic inflammation (and FCN1 protein activity) [14], and type 1 diabetes risk [15], respectively. All four *trans*-eQTL associations observed for the FCN1 region (represented by rs10120023) were substantially reduced after adjustment for FCN1 expression (ILMN_1668063) (Fig. 3). Similar results for GATA2 and RPS26 are shown in Figure S5.

Among the 245 *trans*-eQTL associations for which no potential mediator was identified in the *cis*-eQTL analysis, we selected the probe with the strongest association to the lead *trans*-eSNP and conducted mediation analysis. However, little evidence of mediation was observed (Figure S6).

### “Partial mediation” can be due to measurement error and low LD

**Figure 4:**
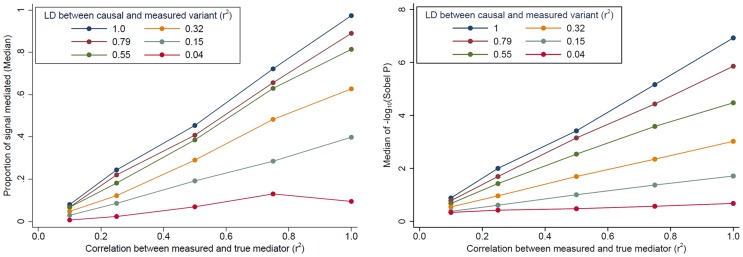
The impact of *cis*-transcript measurement error and LD on evidence for mediation based on simulated data. When the effect of a genetic variant on a *trans*-gene is completely mediated by a *cis*-transcript, evidence of mediation decreases, in terms of the “proportion of signal that is mediated” (left) and the Sobel P (right), as measurement error increases and as the LD between the causal and measured variant decreases.

Evidence for mediation is often observed as “partial” mediation, as the *trans*-eQTL association is not completely eliminated after adjustment for a *cis*-transcript. To aid our interpretation of partial mediation, we simulated *cis*- and *trans*-eQTL expression data based on real genotype data assuming complete mediation (see Methods). Our simulations show that evidence for mediation (in terms of the “proportion mediated” and the Sobel P) decreases as LD between the causal variant and the measured variant decreases and as measurement error increases (measured as the correlation between the true mediator and our measurement of the mediator) (Fig. 4). In other words, even when *trans*-eQTL associations are fully mediated by a *cis*-transcript, evidence for mediation will be detected as “partial mediation” when there is measurement error for the mediating probe and/or imperfect LD between the causal variant and the measured variant under analysis.

### The effect of *cis*-*trans* confounding on mediation analysis

**Figure 5:**
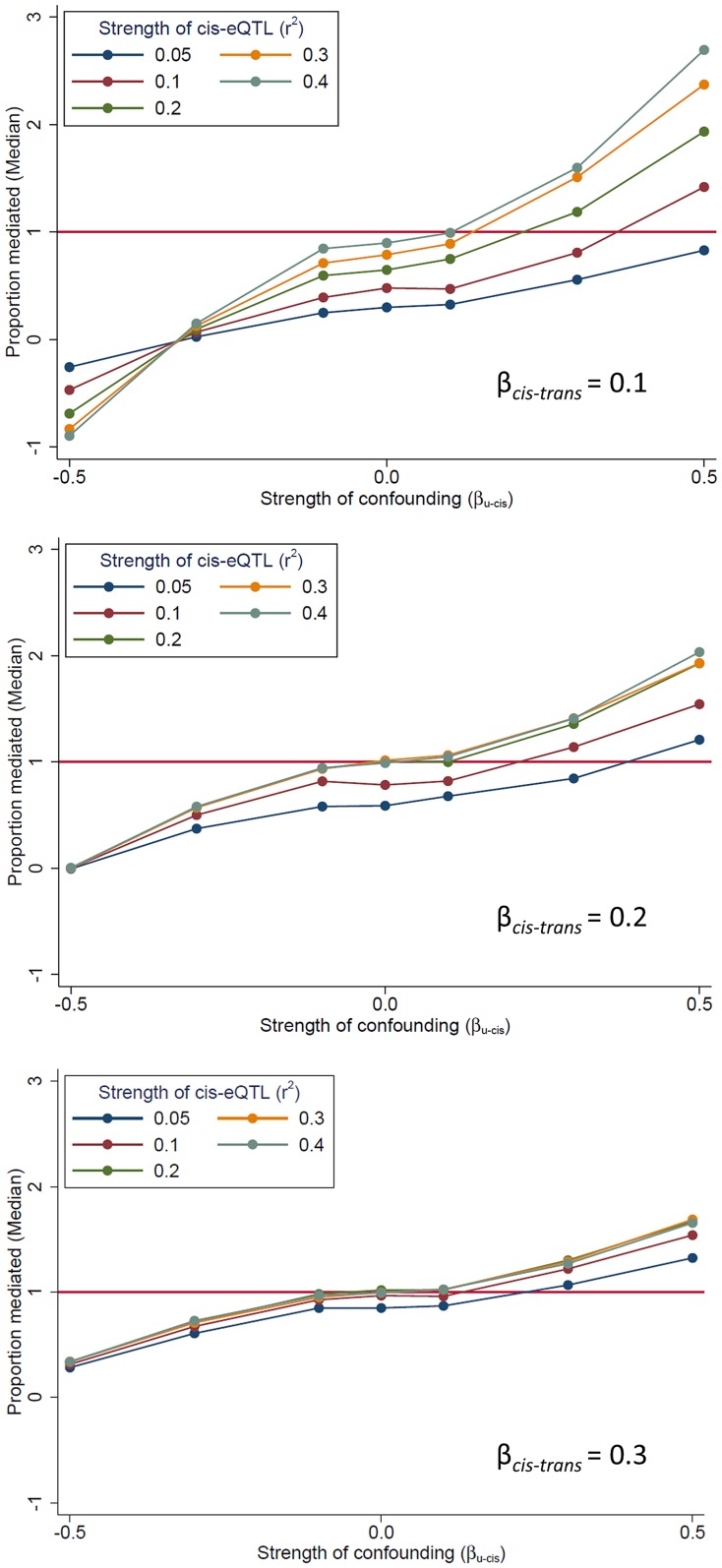
The presence of unmeasured confounding of the “*cis*-mediator”-“*trans*-gene” relationship can bias mediation estimates. We use simulated data to demonstrate that an unobserved variable U which affects both the *cis*-mediator by (effect size of β_U-*cis*_) and the *trans-*gene (effect size of β_U-trams_  =  |β_U-*cis*_|) can bias the estimate of the “direct effect” of the SNP on the *trans*-gene (β_adj_), resulting in bias in the estimate of the proportion of the *trans*-eQTL effect that is mediated (β - β_adj_)/β). All simulated scenarios are “complete mediation”, so the true value of “proportion mediated” is 1.0 (horizontal line). Mediation scenarios are categories according to the strength of the *cis*-eQTL, in terms of r^2^, and the strength of the effect of the *cis*-gene on the *trans*-gene (β*_cis_*_-*trans*_). The SNP is coded as the number of alleles which increase the abundance of the *cis*-transcript.

In our mediation analysis, the estimate of the mediation proportion is less than zero, and occasionally greater than 1 (Fig. 2), a somewhat counter-intuitive finding that suggests the presence of bias. One potential source of bias is “mediator-outcome” confounding [16], which occurs when an unobserved variable (or set of variables) affects both the *cis*-mediator and the *trans*-transcript. In this scenario, the estimate of association between the SNP and the *trans-*gene when adjusting for the potential mediator (i.e., the “direct effect” of the SNP on the *trans*-gene, β_adj_) will be biased. When *cis*-*trans* confounding is absent, the direct effect under full mediation should be zero (β_adj_ = 0; percent mediation  = 100%). Using simulated data, we demonstrate the effect of this bias on the estimate of proportion of the *trans*-eQTL effect that is mediated (β - β_adj_)/β) (Fig. 5). This bias can go in either direction, depending on the directions of the effects of the confounder with the *cis*-mediator, the confounder on the *trans*-transcript, and the non-reference allele (in our case, the minor allele) on the *cis*-transcript (Figure S7). The magnitude of the bias depends on the strength of confounding and the effect of the *cis*-gene on the *trans*-gene. Thus, there is no expectation regarding the direction in which this bias should affect the estimate of β_adj_. Exceptionally strong bias has the potential to qualitatively change the results of mediation analysis, such large changes in the direction or substantial strengthening of a *trans*-eQTL association after adjustment for a potential *cis*-mediator, but we observe very few examples of these phenomena in our data (see below).

### Mediation can be detected when the true mediator is not measured

In addition to *cis*-*trans* confounding, bias can arise when the analyzed *cis*-transcript is not the true mediator, but is correlated with the true mediator. More specifically, evidence for mediation will be observed if the transcript used for mediation analysis is influenced by a *cis*-variant that is in LD with the causal *trans*-eSNP *and* is correlated with the true mediator, due either to confounding (due to an unobserved transcript or factor) or a causal relationship between the analyzed transcript and the true mediator (Figure S8 and Figure S9). When the causal relationships shown in Figure S8B and Figure S8C are positive effects (producing positive correlations), *and* the LD between the expression-increasing alleles is positive, the adjusting for the selected transcript will attenuate the *trans*-eQTL association. However, when both positive and negative relationships are present, adjusting for the selected transcript can increase the magnitude of the *trans*-association (Figure S9A and Figure S9B). Thus, even when the true mediator is not measured, it is still possible to obtain indirect evidence of that a *trans-*eQTLs is attributable to *cis-*mediation.

In contrast, when an unobserved variable influences both the selected *cis*-transcript and the *trans*-gene (Figure S8D), evidence of mediation can be falsely detected, and similar to *cis*-*trans* confounding, the estimate can be biased in either direction (Figure S9C), depending on the direction of the effects of the confounder and the positivity/negativity of the LD between expression-increasing alleles.

### Adjusting for a potential *cis*-mediator can strengthen or change the direction of a *trans*-association

We observe several instances in which adjusting for a potentially-mediating transcript substantially strengthened or reversed the direction of the *trans*-association (Sobel P<10^−5^) (Table S3). As noted in the above sections, this estimate could potentially be biased due to exceptionally strong *cis*-*trans* confounding. However, additional causal diagrams that are consistent with this phenomenon are shown in Figure S10. In the first scenario, a causal *trans*-eQTL variant affects a *trans*-gene though multiple *cis*-mediators. In the second, two causal *trans*-eQTL SNPs are in LD, and each affects the same *trans*-gene, through two different *cis*-mediators. In order to determine if these proposed scenarios potentially explain our two most striking examples of these phenomena, we regressed the *trans*-gene on the *trans*-eSNP, adjusting for all measured *cis*-transcripts correlated with the *trans*-eSNP (Table S3). For these *trans*-eQTLs, adjusting for additional transcripts did not substantially attenuate the *trans*-association, suggesting that these “direction changes” are due to unmeasured mediators or unobserved confounding variables.

### Characteristics of *trans*-eQTLs that do not show strong evidence of mediation

For>140 of our 189 significant *trans*-/*cis*-eSNPs (**Fig. 2A**), the Sobel P is>10^−5^ and the “mediation proportion” is distributed around zero. While it is difficult to identify specific examples of true mediation among this group, we hypothesize that non-uniformity of the Sobel P-value distribution in this range is likely due to a mixture of true mediation, bias due to confounding of the *cis*-*trans* association, and correlation between the true (unmeasured) mediator and the probe selected for mediation analysis. These phenomena are described in detail in the sections above, and the latter two phenomena can result in both positive and negative bias for the estimate of the “mediation proportion”.

Among our observed *trans*-eQTLs, those that do not show mediation are, on average, further from transcription start sites or end sites (TSS or TES) as compared to those that show mediation (Table S4), suggesting that some *trans*-eQTLs do not influence the expression of nearby protein-coding genes. When considering only *trans*-eSNPs that lie <30 kb from a TSS or TES, those that do not show mediation are more likely to lie near a gene for which we do not have expression data (Table S4), suggesting that a substantial number of the true mediators for these *trans*-eQTLs are not represented by probes in our dataset. *Trans*-eSNPs showing no mediation were somewhat more likely to tag (r^2^>0.7) non-synonymous SNPs than non-mediated *trans*-eSNPs, indicating that coding changes may mediate the effects of *trans*-eQTLs for which we could not identify clear mediators (Table S4). A similar pattern was not observed for splice-modifying SNPs.

### Replication of *trans*-eQTLs

A total of 43 of the 419 (10%) unique gene-level *trans*-eQTLs observed among our Bangladeshi participants (FDR of 0.05) have been observed in prior *trans*-eQTL studies using RNA from blood cells (peripheral or transformed) from participants of primarily European ancestry [5], [7], [11], [17]. Among the 2,493 *trans*-eQTLs with P<10^−7^, 59 have been observed in prior studies (Table S5). Probability of replication depended strongly on P-value, with 27% of our findings with P<10^−15^ replicating, 7% with P between 8.4×10^−9^ and 10^−15^, and 1% with P between 10^−7^ and 8.4×10^−9^. For *trans*-eQTLs passing the FDR threshold, there was not strong evidence that those showing evidence of mediation were more likely to replicate than those that did not show evidence of mediation, however, for *trans*-eQTLs not passing the FDR threshold (P>8.4×10^−9^ but P<10^−7^), a higher percentage of replication was observed among mediated *trans*-eQTLs, although there were only 16 of mediation among this group (Table S6).

### Replication of mediation results

Using data from two independent cohorts of European Ancestry, the Groningen (n = 1,240) and Estonian EGCUT (n = 891) cohorts [11], we attempted to replicate our mediation signals. In these cohorts, complete data on the lead SNP, *cis*-probe, and *trans*-probe (based on RNA from whole blood) were available for 21 of the mediated *trans*-eQTL associations, and only one of these showed strong evidence of being a *trans*-eQTL in both cohorts. For this eQTL (rs6785206, associated with GATA2 in *cis* and CLC in *trans*), we observed evidence of mediation in both cohorts, with the *trans*-eQTL association reduced in magnitude by 21% and 38%, respectively, after adjusting for the *cis*-mediator. Six additional *trans*-eQTL associations were replicated in one or the two cohorts, and we observed evidence consistent with cis-mediation for five of the six (Table S7).

## Discussion

In this work, we have conducted a comprehensive analysis of *cis*-mediation underlying *trans*-eQTLs using data from the first large genome-wide eQTL study of South Asian individuals. Approximately 44% of all *trans*-eQTLs detected at an FDR of 0.05 also showed evidence of being a *cis*-eQTL, enabling analysis potential mediation by *cis*-transcripts. Among analyzed *trans*-eQTLs, ∼21% showed strong evidence of mediation by a measured *cis*-transcript. Analysis of simulated data demonstrated that partial rather than complete mediation will be detected in the presence of (1) measurement error for mediating transcripts and (2) imperfect LD between measured SNPs and the causal variants. Simulations also demonstrate that *cis*-*trans* confounding can bias estimates obtained from mediation analysis, while correlations among neighboring *cis*-transcripts, can enable detection of mediation when the true mediator is unmeasured. Observing evidence of mediation was more likely for *trans*-eQTLs with smaller P-values and when the lead *trans*-eSNP was in strong LD with the lead *cis*-eSNP for the potentially-mediating transcript. Demonstration of *cis*-mediation for observed *trans*-eQTLs provides a form of validation, a clear biological mechanism, and an approach for enhancing future *trans*-eQTL discovery.

Among our 434 significant *trans*-eQTL associations, we lacked data on potential mediators for 245 *trans*-associations (i.e., the lead *trans*-eSNP was not identified as a *cis*-eSNP in genome-wide *cis*-eQTL analyses). This lack of data on potential mediators could be due to several factors. First, many mediators may be unmeasured or excluded as a consequence of QC (Table S4). Second, some *trans*-eQTL effects may not be mediated by expression of a *cis*-transcript. For example, a *trans* effect could be due to variation in the coding sequence of a regulatory factor (Table S4), physical inter-chromosomal interactions, non-coding RNA, or other mechanisms that do not entail mediation by *cis*-expression of a protein-coding gene. Third, some *trans*-eQTLs may be false positives. This is likely the case for many *trans*-eQTLs of marginal significance (5×10^−9^<P<10^−7^), which are less likely to be *cis*-eQTLs than FDR-significant *trans*-eQTLs. However, even for highly-significant *trans*-eQTLs (P<10^−15^), ∼38% lack data on a potential *cis*-mediator (Table 2). Fourth, it is possible that *trans*-eQTLs may be due to *cis* effects that are detectable as very weak associations in our dataset; however, our mediation analysis for *trans*-eSNPs that were not identified as *cis*-eSNPs did not provide strong evidence for this hypothesis (Figure S6). All of these phenomena are possible explanations for the substantial number of *trans*-eQTLs for which we lack data on a potential cis-mediator.

Our working hypothesis is that a substantial fraction of *trans*-eQTLs are fully-mediated by a transcript that is regulated in *cis* by the causal *trans*-eQTL variant (Fig. 1). While we did not observe complete mediation for most observed *trans*-eQTLs, we demonstrate that full mediation will be observed as partial mediation in the presence of mediator measurement error and/or imperfect LD between the causal variant and the variant used for analysis. Measurement error and imperfect LD are typically present in eQTL studies; thus, full mediation will frequently be observed as partial mediation. Factors that contribute to measurement error include: experimental error, cell type-specific eQTLs in the presence of cell mixtures, stochastic or temporal variability in expression, and non-specific measurement of the mediating transcript(s) (i.e., some probes bind multiple isoforms). Observing partial mediation may also be due, in part, to the Winner's curse, as *trans*-eQTL associations that are overestimated may not be fully explainable by a *cis*-mediator. For the *trans*-eQTL that lacked clear evidence of mediation, potential explanations include: analyzing a *cis*-transcript that is not the true mediator (perhaps due to missing data), low power due to measurement error, or a false positive *trans*-eQTL.

In this work, we observe a subset of *trans*-eQTLs that are clearly attenuated after adjustment for a *cis*-mediator (i.e., mediation proportion>0 and Sobel P<10^−5^), as expected based on the mediation hypothesis (Fig. 2A). However, for the remaining *trans*-eQTLs, the mediation proportion estimates are scattered around zero (i.e., the *trans* association often gets somewhat stronger after adjustment) and the Sobel P distribution is non-uniform. We hypothesize that many of these “significant” estimates are due to a mixture of true mediation and the various sources of potential bias we describe in this work, including *cis*-*trans* confounding and correlation between the true (unmeasured) mediator and the transcript selected for mediation analysis. These types of bias have no expectation regarding directionality, so a distribution of mediation proportions that includes zero is expected.

Potential confounders of the *cis*-*trans* association include demographic and environmental factors, as well as a wide-array of biological phenomenon, some of which may be captured by measured expression features. Omitting such variables from the regression model can bias the estimates of the “direct effect” of the SNP on the *trans*-gene and the “mediation proportion”. The direction of this bias will depend on the direction of the effects for the omitted confounder(s). We attempted to control for potential confounding factors in this work using only principle components adjustment (see methods), but this limitation did not prevent us from detecting many examples of *cis*-mediation. However, confounding bias is likely to prevent detection of weaker mediation signals. Because genome-wide expression data contains very large numbers of correlated genes (too many to adjust for individually), additional research is needed to develop methods for comprehensive adjustment for *cis*-*trans* confounding in analyses of mediation in the genome-wide setting.

Few prior studies have assessed *cis*-mediation for *trans*-eQTL associations at the genome-wide level. Jian et al. described the use of mediation analysis in order to identify eQTLs for CYP2D6 activity [18]. Battle, et al. [19] used RNA sequencing data from whole blood on 922 genotyped individuals to characterize the effects of regulatory variation on transcriptome diversity. They observed 138 genes regulated by *trans*-variants and 76 *trans*-eQTL SNPs that were associated with expression of a proximal gene. Using a likelihood-based approach [20], [21], Battle et al. reported that 85% of identified *trans* effects were mediated by *cis* transcripts, but with only 4% showing evidence of “full mediation” and the remaining 81% showing evidence of partial mediation. However, the likelihood-based test for partial mediation used by Battle, et al. is also based on a regression the *trans*-gene on the SNP and the *cis*-mediator, and is therefore also prone to confounding biases caused by unobserved variables.

The number of *trans*-eQTLs observed in this South Asian population is somewhat larger than prior studies of similar sample size [5], [6], [11]. Prior studies have also noted low rates of replication for *trans*-eQTLs across studies, even for studies of similar ancestry [5], [9]. For example, 46 of the 130 *trans*-eQTLs observed by Fehrmann et al. (in whole blood among 1,469 samples) could be replicated in an eQTL study of monocyte RNA at P<10^−5^ among 1,490 samples [6]. Difficulties in replication have also been observed in *trans*-eQTL studies of mice [22]. Replication was markedly better in a recent *trans*-eQTL meta-analysis, presumably due in part to large sample size (n>5,000) and a focus on trait-associated SNPs [11]. Several factors may contribute to the low rates of replication of our observed *trans*-eQTLs in prior studies. First, differences between population, both genetic and environmental may impact trans-eQTL patterns. Second, there are differences in RNA source, as prior *trans*-eQTL studies used whole blood or monocytes, while our source is PBMCs, consisting of monocytes (∼15%), T lymphocytes (∼65%), and B lymphocytes (∼20%), representing ∼35% of peripheral white blood cells. Third, we lack complete lists of *trans*-eQTL for replications purposes, as we are limited to examining lists of only the strongest *trans*-eQTL associations provided by the authors of prior papers.

Mediation analysis is an attractive method for characterizing observed *trans*-eQTL associations for several reasons. First, it provides putative regulatory mechanisms for observed *trans*-eQTLs, potentially enhancing our understanding of disease-associated variants and human disease biology. Second, evidence of mediation provides a form of validation for *trans*-eQTLs. Independent replication (using the same cell type and population) is the ideal form of validation, but such data sources are not always available. Third, detecting mediation is methodologically straightforward, requiring only conditional linear regression techniques and simple equations for estimating effects and significance (see methods) [23]. Fourth, evidence of mediation could potentially be used as weights in integrative analyses or as priors in Bayesian analyses to enhance discovery of *trans*-eQTLs that have measured mediators. Mendelian randomization (i.e., instrumental variable (IV)) approaches could also be considered as a complimentary approach, in which *cis*-eSNPs are used as IVs for *cis*-transcripts and which are then screened, genome-wide, for effects on expression of *trans*-genes.

Our ability to detect *cis*-mediation will be enhanced by using whole-transcriptome RNA-sequencing data, which capture the vast majority of transcribed sequences, better reflecting the full complexity of the transcriptome. Array-based platforms capture only a fraction of transcribed sequences and probe-based measures often reflect multiple isoforms of a gene. RNA-sequencing can also provide enhance measurement precision for mediating transcripts, as would cell-type specific RNA sources. Very high-density genotype data will also enhance mediation analysis, as statistical evidence of mediation will be stronger when causal variants or strong tagging variant are directly measured.

In terms of sample size, this is the largest *trans*-eQTL study of humans to date that analyzes genome-wide variants in an agnostic fashion. An additional strength of this study is the rapid time between the blood draw and the extraction and processing of RNA. While this is often not reported in eQTLs studies conducted in the epidemiological setting, our processing protocol is excellent for studies of this size, especially for a low-resource setting such as Bangladesh. In addition, this is the first eQTL study conducted in a South Asian population. A recent multi-population eQTL study included U.S. residents of Indian ancestry [3]; however, the sample was relatively small, and RNA was obtained from lymphoblastoid cells lines and was thus prone to the effects of transformation with Epstein-Barr virus.

In conclusion, we have described *cis*- and *trans*-eQTLs in a large sample of South Asians and used mediation analysis to provide evidence that *cis*-mediation is often observed for *trans*-eQTLs in humans. In addition, using simulated data, we demonstrate how unobserved confounding variables and incorrect mediator selection can bias mediation estimates. Mediation analysis will be useful for validation and discovery of *trans*-eQTLs (especially when appropriate data for replication is not available) and is a valuable tool for enhancing our understanding of the biological and regulatory mechanism underlying *trans*-eQTLs.

## Methods

### Study participants

Subjects genotyped for this work were participants in the Bangladesh Vitamin E and Selenium Trial (BEST) [24]. BEST is a 2×2 factorial randomized chemoprevention trial evaluating the long-term effects of vitamin E and selenium supplementation on non-melanoma skin cancer risk among 7,000 individuals with arsenic-related skin lesions living in Araihazar, Matlab, and surrounding areas. Participants included in this work are a subset of BEST participants from Araihazar that have available data on genome-wide SNPs and array-based expression measures (described below).

### SNP data and imputation

DNA extraction was carried out from the whole blood using the QIAamp 96 DNA Blood Kit (cat # 51161) from Qiagen, Valencia, USA. Concentration and quality of all extracted DNA were assessed using Nanodrop 1000. As starting material, 250 ng of DNA was used on the Illumina Infinium HD SNP array according to Illumina's protocol. Samples were processed on HumanCytoSNP-12 v2.1 chips with 299,140 markers and read on the BeadArray Reader. Image data was processed in BeadStudio software to generate genotype calls.

Quality control was conducted as described previously for a larger sample of 5,499 individuals typed for 299,140 SNPs [25], [26]. First, we removed DNA samples with very poor call rates (<90%; n = 8) and SNPs that were poorly called (<90%) or monomorphic (n = 38,753). Individuals with gender mismatches were removed (n = 79), as were technical replicate DNA samples run to assure high genotyping accuracy (n = 53). No individuals had outlying autosomal heterozygosity or inbreeding values. After inspecting distributions of SNP and samples call rates, we excluded samples with call rates <97% (n = 5) and SNPs with call rates <95% (n = 1,045). SNPs with HWE p-values<10^−10^ were excluded (n = 1,045). This QC resulted in 5,499 individuals with high-quality genotype data for 257,747 SNPs. The MaCH software [27] was used to conduct genotype imputation using HapMap3 GIH reference haplotypes. Only high-quality imputed SNPs (imputation r^2^>0.5) with SNPs with MAF>0.05 were included in this analysis. A subset 1,799 individuals with available data on array-based expression measures was used for this project

### Gene expression data

RNA was extracted from PBMCs, preserved in buffer RLT, and stored at −86°C using RNeasy Micro Kit (cat# 74004) from Qiagen, Valencia, USA. Concentration and quality of RNA samples were assessed on Nanodrop 1000. cRNA synthesis was done from 250 ng of RNA using Illumina TotalPrep 96 RNA Amplification kit. As recommended by Illumina we used 750 ng of cRNA on HumanHT-12-v4 for gene expression. Expression data were quantile normalized and log_2_ transformed. The chip contains a total of 47,231 probes covering 31,335 genes. There were 1,825 unique individuals in both expression data and SNP data. For the vast majority of participants, between 30% and 47% of probes had detection P values <0.05. However, 26 individuals had>30% of probes with detection p-value <0.05, and these outlying individuals were excluded from the analysis, leaving an analysis sample size of 1,799. For this analysis, no probes were excluded based on detection P-values.

### Identification of probes that map to a single gene

To ensure each probe mapped uniquely to a single gene, we aligned the Illumina probe sequences to all transcriptome sequences contained in both the knownGeneMrna and the knownGeneTxMrna tables from the UCSC Genome Browser (version GRCh37/hg19). Probe sequences were aligned using BLAST, as recommended by Barbosa-Morais et al. [28]: (blastn -dust no -evalue 10e-6). This resulted in alignments between 38,924 probes and 66,864 transcripts. From these alignments, we selected un-gapped alignments with up to 2 mismatches, as recommended by Barbosa-Morais et al., resulting in alignments between 35,202 probes and 61,350 transcripts. We then determined which transcripts (i.e., isoforms) belonged to the same gene using the knownIsoforms table from UCSC genome browser, which resulted in 35,202 probes mapped to 23,419 isoform clusters (i.e., genes). We did not disqualify probes that mapped to several isoforms of the same gene, provided they did not map to isoforms of any second gene. After excluding probes that mapped to multiple genes, we identified 31,853 probes were specific to 20,143 genes.

### Exclusion of probes that bind to sequences containing SNPs

For the 31,853 specific probes, we obtained absolute genomic coordinates from the UCSC knownGene table, which contains genomic coordinates for all transcripts in knownGeneMrna/knownGeneTxMrna, including gaps introduced by introns. We referred to the UCSC snp135Common table to count the number of SNPs in each probe, according to the probe's genomic coordinates. Out of the 31,853 specific probes, 8,880 probes contained one or more SNPs, and these were excluded from all *cis*-eQTL analyses. Of these, the majority (6,194 probes) only contained a single SNP.

### Expression data processing

We used probe-level data for this analysis (as opposed to combining probe data into gene-level expression traits), primarily because some probes bind specific isoforms of a transcript that are not detected by other probes that target the same gene. Probe intensity values were log-transformed. Batch effects (22 batches total, representing 22 96-well plates) were assessed using the empirical Bayes framework implemented in the Surrogate Variable Analysis (SVA) software package (ComBat) [29]. SVA did not detect any significant surrogate variables, thus we used principle components (PC) analysis to estimate 100 PCs that were subsequently considered as potential latent variables that may represent variability attributable to technical (i.e., non-biological) factors.

### Statistical methods for eQTL analysis

All expression values were log-transformed prior to analysis. Linear regression, as implemented in the matrix-eQTL software package [30] was used to conduct genome-wide *cis*- and *trans*-eQTL analyses. *Cis* associations were tested for SNPs and probes <1 Mb apart (i.e., a 2Mb window centered on each SNP). *Trans*-associations were tested for all inter-chromosomal SNP-probe pairs, as well as for intra-chromosomal SNPs-probe pairs>10 Mb apart. For both *cis* and *trans* analyses, we used an FDR of 0.05 to report the significant associations, as calculated by the matrix-eQTL software. We generated data on 100 PCs, and conducted *cis*-eQTL analyses multiple times, adjusting for 20, 40, 60, 80, and 100 PCs. The number of *cis*-eQTLs detected increased as we adjusted for additional PCs, but the increase in power was very small for 100 PCs as compared to 80, so we elected to adjust for 80 PCs in the *cis*-eQTL analysis. For the *trans*-eQTL analysis, we only adjusted for the 14 (of the 80) PCs that were not associated with any SNP at a P-value>5×10^−8^. Genome-wide *trans*-eQTL analyses showed little evidence of inflation due to population structure of genotyping artifacts (Figure S11), consistent with our prior work demonstrating little evidence of population structure among our study participants. Lead SNPs fear each eQTL were defined as the SNP with the smallest P-value for an expression trait, with *trans*-associations>5Mb apart considered independent *trans*-eQTL associations.

### Assessment of off target binding for probes involved in *trans*-eQTLs

Among our observed *trans*-eQTL associations with P<10^−7^, we sought to eliminate false positives due to loose, off-target binding of the expression probe near the correlated SNP. A localized high-sensitivity BLAST was performed (blastn -dust no -evalue 1000). For each instance of BLAST, the query was the sequence of the expression probe from our list of *trans*-eQTLs, and the target was the genome sequence from a 4Mb window centered on the corresponding SNP (hg19, retrieved from UCSC). Note that our initial probe QC used a lower-sensitivity BLAST with a much larger query and target sets (i.e.: all HT-12 probes and all sequences UCSC's knownGeneMrna, knownGeneTxMrna). After identifying putative *trans*-eQTL associations, smaller query and target sets could be selected for a higher-sensitivity BLAST. This two-stage approach was also used by Fehrmann et al. [5]. From the BLAST results, we accepted alignments with: alignment length> = 15bp; or alignment length> = 20 and number of mismatches < = 1; or alignment length> = 30 and number of mismatches < = 2; or alignment length> = 50 and number of mismatches < = 15.

### Enrichment analysis

Trait-associated SNPs were selected from the NHGRI's GWAS catalog based on a reported P<5×10^−8^. The resulting list of SNPs was pruned to eliminate SNPs with high LD (no pair-wise r^2^>0.3). For the GWAS enrichment analysis, we compared the catalog SNPs with a reported P<5×10^−8^ to catalog SNPs with P>5×10^−8^, a method previously used by Westra et al. [11]. For this analysis, a Fisher's exact test was used to assess significance of enrichment. For the *cis/trans* enrichment analysis, random sets of SNPs were extracted from our dataset matched to our set of trait-associated SNPs based on MAF (10 categories) and distance to transcription start site (10 bins). Empirical P-values were estimated using 1,000 replicate datasets.

### Mediation analysis

To identify *trans*-eQTLs showing evidence of mediation, we restricted to those lead *trans*-eSNPs (P<10^−7^) which has at least one associated nearby (<1 Mb) probe (*cis*-probe) with association (P<2.2×10^−3^, the P threshold used for the *cis*-eQTL analysis). For *trans*-eSNPs associated with multiple *cis*-probes, we selected the associated *cis*-probe whose lead *cis*-eSNP was in strongest LD with the lead *trans*-SNP. Mediation analysis was conducted as follows: For lead *trans*-eSNPs that were associated with a *cis* probe, the *trans*-association was re-estimated, adjusting for expression of the local transcript measured by the probe. The difference between the beta coefficients for the *trans*-association before and after adjustment for the *cis* probe was expressed as the “proportion of the total effect that is mediated” (i.e., % mediation), calculated as (β_unadj_ – β_adj_)/β_unadj_ [23], with β_unadj_ and β_adj_ known as the *total effect* the *direct effect*, respectively. The Sobel P-value for mediation [31] was calculated by first estimating the *cis*-adjusted *trans*-association for the lead *trans*-eSNP:
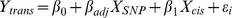



We then estimated the *trans*-eSNP's association with the probe in *cis* (the potentially mediating probe):
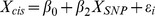



The P-value was then estimated by comparing this following t statistic to a normal distribution:
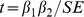


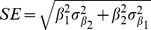



where SE is the pooled standard error term calculated from the above beta coefficients and their variances. β_1_ β_2_ is often referred to as the *indirect effect*.

### Simulations to assess why “partial” mediation is observed

Using real genotype data for all 1,799 participants, we selected a causal variant for simulation purposes and generated data on a *cis*-transcript (standard normal distribution) influenced by the causal variant (R^2^ = 0.1) and a *trans*-transcript (standard normal distribution) influenced by the *cis*-transcript (β = 0.2). We introduced measurement error by adding normally-distributed error components to both the *cis*- and *trans*-transcripts. Standard deviations for these components were chosen to produce specific r^2^ values (1.00, 0.75, 0.50, 0.25, and 0.10) for the correlation between the true mediator and the measure transcript, with lower values reflecting higher error. For each measurement error scenario, 500 datasets were simulated, and analyses were conducting using variants with a wide range of LD (i.e., r^2^ values) with the true causal variant.

### Simulations to assess the impact of confounding between *cis*- and *trans*-transcripts

In order to assess the impact of confounding between a *cis*-mediating transcript and a *trans*-gene involved in a *trans*-eQTL association, we generated data similar to that described above, but introduced an “unobserved” variable (U) that affects both the *cis*- and *trans*-transcripts. We varied the strength of the *cis*-eQTL effect in terms of its r^2^ (0.05 to 0.4), the strength of the effect of the *cis*-transcript on the *trans*-transcript (0.1, 0.2, and 0.3), and the strength of the confounding relationship in terms of the effects of U on the *cis*-transcript (β_U-*cis*_) and the *trans-*transcript (β_U-trams_  =  |β_U-*cis*_|). The SNP was coded as the number of alleles that increase the abundance of the *cis*-transcript.

### Simulations to assess the consequences of selecting the wrong mediator

In order to assess the impact of selecting a *cis*-transcript for mediation analysis that is not the true mediator, we conducted similar simulations as those described above, but generated data on an additional transcript influenced by a variant near the causal variant for the true mediating transcript. We first selected several SNPs near the selected causal variant for the primary *cis*-/*trans*-eQTL with a wide range of LD r^2^ values, and we then treated these variants as causal variants for a second eQTL association for a different transcript that does not affect the *trans*-transcript. In the simplest scenario, we simulated data with no dependency between the true cis-mediator and the selected transcript other than correlation due to LD between the causal variants that influence their expression (Figure S8A). We then introduced correlation between the two *cis*-transcripts using several different approaches. First, we introduced confounding by an unmeasured factor, using effect sizes of 0.3 and 0.5 on both transcripts to represent “weak” and “strong” confounding (Figure S8B). We also introduced “negative confounding”, in which the effect of the unmeasured confounder was positive for one *cis*-transcript and negative for the other. Second, we introduced an effect of the true cis-mediator on the selected transcript, exploring both positive and negative effect (beta  = 0.25 and −0.25), as well as the reverse causal relationship where the selected transcript affects the true cis-mediator (beta  = 0.25 and −0.25). Lastly, we introduced an unmeasured confounding factor affecting both the *trans*-gene and the selected *cis*-transcript (Figure S8D).

### Replication of *trans*-eQTLs based on previous eQTL studies

Data for replicating observed *trans*-eQTLs was obtained from several prior *trans*-eQTL studies using a RNA extracted from peripheral blood or subtypes of white blood cells [5], [7], [11]. Consensus *trans*-eQTLs from HapMap lymphoblastoid cells line studies were also used for replication purposes [17]. Considering multiple genotyping and expression platforms were used across these studies, replication as defined as *trans*-associations which involve the same expressed gene (based on HUGO gene symbol) and the SNPs in the same genomic region (<500 kb apart).

### Ethics and data sharing statements

This research as approved by the Institutional Review Boards of The University of Chicago, Columbia University, and the Bangladesh Medical Research Council, and all study participants provided informed consent. Summary statistics for the *cis*- and *trans*-eQTL analyses as well as the mediation analysis are available at http://doi.org/10.5061/dryad.tp097.

## Supporting Information

Figure S1 Scatter plot for all observed *trans*-eQTLs at P-value thresholds of 10^−7^, 8×10^−9^, and 10^−15^. *Trans*-eQTLs with strong evidence of Mediation (Sobel P<10^−5^ and mediation proportion>0) are shown in red.
(TIF)

Figure S2 Enrichment for *cis*-eQTLs among trait-associated SNPs.
(TIF)

Figure S3 Enrichment for *trans*-eQTLs among trait-associated SNPs.
(TIF)

Figure S4 Enrichment for *trans*-eQTLs among *cis*-eQTL SNPs.
(TIF)

Figure S5 RPS26 and GATA2 expression are the primary mediators of the *trans*-eQTLs located at loci involved in type 1 diabetes risk (12q13.2) and systemic inflammation (9q34.3), respectively. The P-values (left) and beta coefficients (right) for four *trans*-eQTL associations in the RPS26 (top) and GATA2 (bottom) regions are reduced in significance after adjusting for expression of RPS26 and GATA2, respectively.
(TIF)

Figure S6 Little evidence of mediation for *trans*-eQTLs showing weak associations with *cis*-transcripts. The proportion of a *trans*-eQTL mediated by a *cis*-transcript (i.e., the “mediation proportion) is plotted against the −log_10_(Sobel P) for *trans*-eQTLs that were not identified as *cis*-eQTLs in our genome-wide analysis. For the lead eSNP for each of these 245 *trans*-eQTL associations, we selected the strongest associated probe and conducted mediation analysis.
(TIF)

Figure S7 Determinants of the direction of *cis*-*trans* confounding bias for the *trans*-eQTL association after *cis*-mediator adjustment. The direction of bias for the estimate of the “direct effect” (β_adj_) is dependent on the directions of the effects of the confounder (U) on the *cis*-mediator (gene*_cis_*) and *trans*-gene (gene*_trans_*) and the effect of the SNP on the gene*_cis_*.
(TIF)

Figure S8 Causal diagrams representing scenarios in which the true mediator is not used for mediation analysis. These diagrams represent the simulated datasets used to generate effect estimates and P-values shown in S9 Figure. The transcript selected for mediation analysis (gene_s_) may not be a measure of the true mediator (gene_m_). In this case, gene_s_ is a nearby transcript correlated with the causal *trans*-eQTL variant (SNP*_trans_*) due to LD with a nearby causal *cis*-eQTL variant (SNP*_cis_*). Gene_s_ and gene_m_ are correlated due to LD (panel A), but additional correlation can be due to confounding by unobserved factors (U) (panel B) or direct effects of gene_s_ on gene_m_ or vice-versa (panel C). In addition, the gene_s_ may be correlated with the *trans*-gene (gene*_trans_*) due to an unobserved confounder (panel D).
(TIF)

Figure S9 Evidence for mediation can be detected when the true mediator is not measured. According to simulated data described in S8 Figure, if a measure of the true mediator is not included in the analysis, evidence for mediation, in terms of the “proportion mediated” (left) and the Sobel P (right) will be present if the transcript selected for analysis is correlated with the true mediator, either due to confounding (A) or a direct effect (B). Evidence for mediation can be falsely detected due to bias caused by confounding of the relationship between the selected transcript and the *trans*-gene (C). Evidence for mediation will be weaker if LD between the causal *trans*-eQTL variant and the causal *cis*-eQTL variant is low.
(TIF)

Figure S10 Causal diagrams demonstrating scenarios in which adjusting for a single potential mediator may result in a *trans*-eQTL association that is stronger or in the opposite direction of the unadjusted *trans*-eQTL association. In Panel A, a causal *trans*-eQTL variant affects a *trans*-gene though multiple *cis*-mediators. In Panel B, two causal *trans*-eQTL SNPs are in LD, and these SNPs affect a single *trans*-gene, but through two different *cis*-mediators.
(TIF)

Figure S11 Quantile-quantile plot for the genome-wide *trans*-eQTL analysis.
(PNG)

Table S1 Observed *trans*-eQTLs at an FDR of 0.05 (P<8.4×10^−9^)
(XLSX)

Table S2 A) Characteristics of *trans*-eQTL associations (P<8.4×10^−9^) showing evidence of mediation (mediation proportion>0 and Sobel P<10^−5^). B) Characteristics of "suggestive" *trans*-eQTL associations (8.4×10^−9^ <*trans*-P <10^−7^) showing evidence of mediation (mediation proportion>0 and Sobel P<10^−15^).
(XLSX)

Table S3 A) *Trans*-eQTL signals that reverse direction after adjusting for a potential mediator. B) *Trans*-eQTL signals that get stronger after adjusting for a potential mediator.
(XLSX)

Table S4 Characteristics of *trans*-eSNPs stratified by mediation status.
(XLSX)

Table S5 *Trans*-eQTLs observed in this study and in prior studies.
(XLSX)

Table S6 Replication of *trans*-eQTLs by mediation status.
(XLSX)

Table S7 Replication of *cis*-mediation in two independent cohorts.
(XLSX)
