# Supplementary figures and images for: Mediation Analysis Demonstrates That Trans-eQTLs Are Often Explained by Cis-Mediation: A Genome-Wide Analysis among 1,800 South Asians
Source: PLoS Genet. 2014 Dec 4;10(12):e1004818. doi: 10.1371/journal.pgen.1004818 (PMC4256471; doi:10.1371/journal.pgen.1004818)

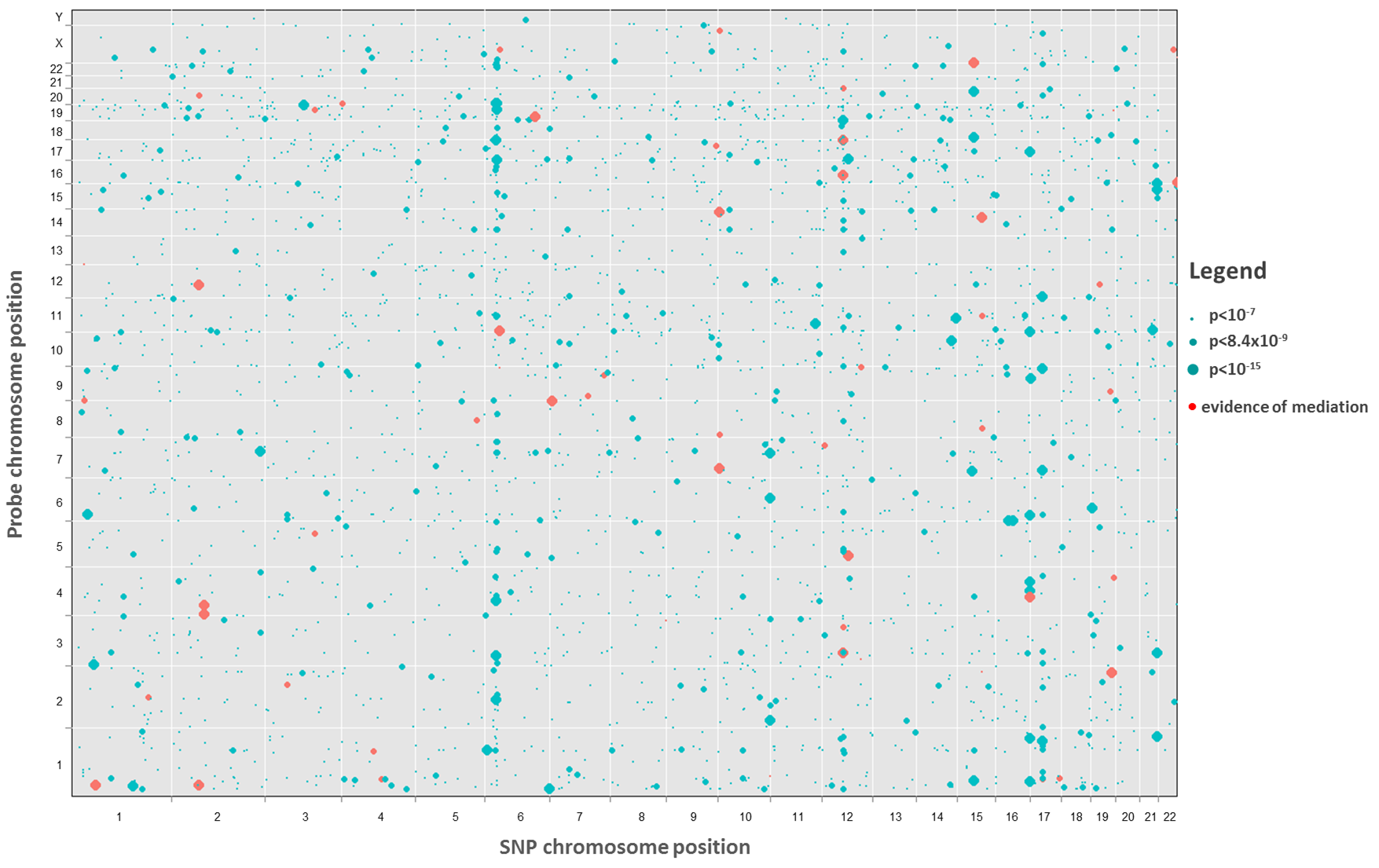

Supplement: Figure S1 — Scatter plot for all observed trans-eQTLs at P-value thresholds of 10−7, 8×10^−9^, and 10−15. Trans-eQTLs with strong evidence of Mediation (Sobel P<10−5 and mediation proportion>0) are shown in red. (TIF) [file pgen.1004818.s001.tif]

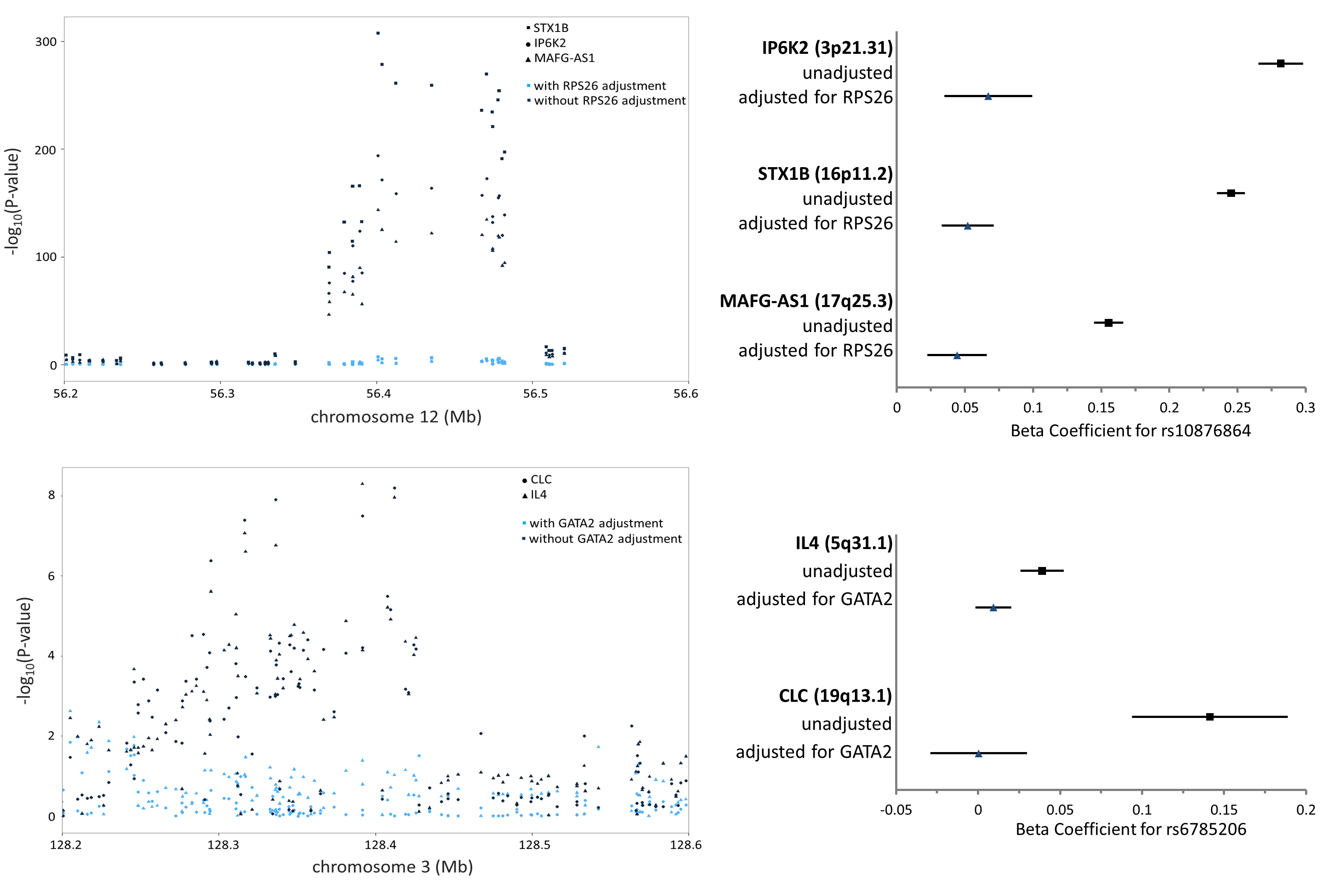

Supplement: Figure S5 — RPS26 and GATA2 expression are the primary mediators of the trans-eQTLs located at loci involved in type 1 diabetes risk (12q13.2) and systemic inflammation (9q34.3), respectively. The P-values (left) and beta coefficients (right) for four trans-eQTL associations in the RPS26 (top) and GATA2 (bottom) regions are reduced in significance after adjusting for expression of RPS26 and GATA2, respectively. (TIF) [file pgen.1004818.s005.tif]

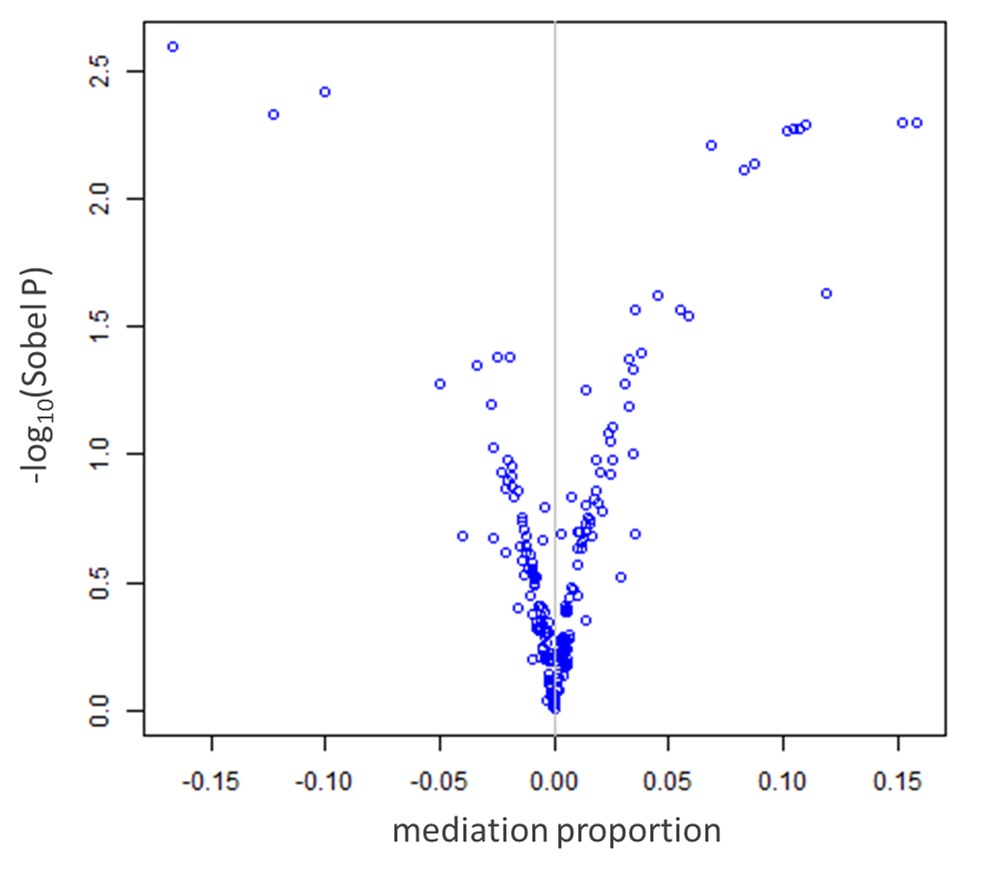

Supplement: Figure S6 — Little evidence of mediation for trans-eQTLs showing weak associations with cis-transcripts. The proportion of a trans-eQTL mediated by a cis-transcript (i.e., the “mediation proportion) is plotted against the −log10(Sobel P) for trans-eQTLs that were not identified as cis-eQTLs in our genome-wide analysis. For the lead eSNP for each of these 245 trans-eQTL associations, we selected the strongest associated probe and conducted mediation analysis. (TIF) [file pgen.1004818.s006.tif]

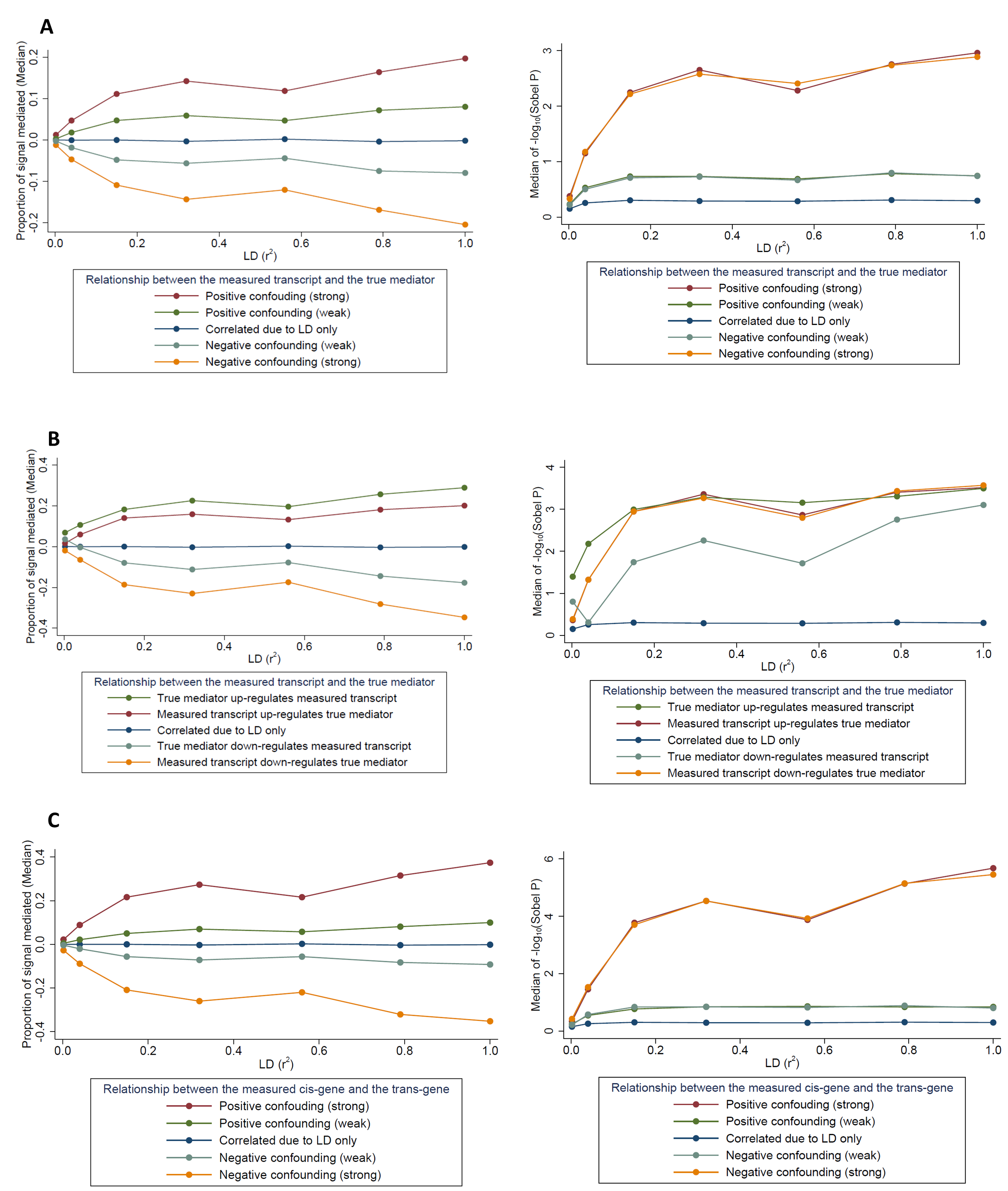

Supplement: Figure S9 — Evidence for mediation can be detected when the true mediator is not measured. According to simulated data described in S8 Figure, if a measure of the true mediator is not included in the analysis, evidence for mediation, in terms of the “proportion mediated” (left) and the Sobel P (right) will be present if the transcript selected for analysis is correlated with the true mediator, either due to confounding (A) or a direct effect (B). Evidence for mediation can be falsely detected due to bias caused by confounding of the relationship between the selected transcript and the trans-gene (C). Evidence for mediation will be weaker if LD between the causal trans-eQTL variant and the causal cis-eQTL variant is low. (TIF) [file pgen.1004818.s009.tif]

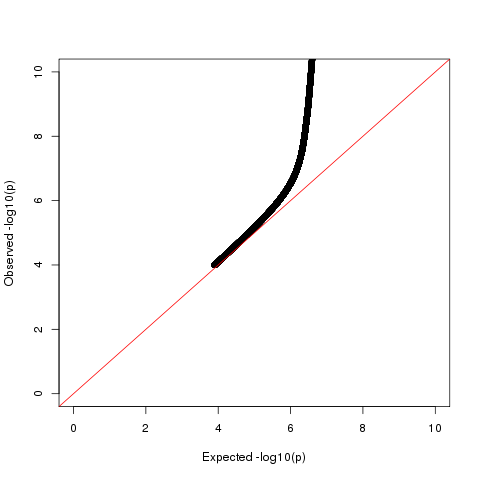

Supplement: Figure S11 — Quantile-quantile plot for the genome-wide trans-eQTL analysis. (PNG) [file pgen.1004818.s011.png]
